# Elevated CD3^low^ double negative T lymphocyte is associated with pneumonia and its severity in pediatric patients

**DOI:** 10.7717/peerj.6114

**Published:** 2018-12-18

**Authors:** Ying Wang, Wenting Lu, Aipeng Li, Zhengyi Sun, Liying Wang

**Affiliations:** 1 Institute of Pediatrics, The First Hospital of Jilin University, Changchun, China; 2 Department of Molecular Biology, Norman Bethune College of Medicine, Jilin University, Changchun, China; 3 Department of Ophthalmology, The First Hospital of Jilin University, Changchun, China

**Keywords:** Children, Pneumonia, Double negative T lymphocyte, CD3, Flow cytometer

## Abstract

**Background:**

Previous studies have shown that the adaptive immunity function of T cells in disease states correlates with CD3 surface expression closely. During routine assessment of TBNK subsets in peripheral blood of pediatric patients by flow cytometry, we noticed that variable expression levels of CD3 on CD3^+^CD4^−^CD8^−^ double-negative T (DNT) lymphocytes in different patients. The objective of this study was to assess the relationship of CD3 expression levels on DNT cells with disease severity.

**Methods:**

In this prospective study, we investigated the frequencies of circulating CD4^−^CD8^−^ DNT cell subsets with CD3^low^ or CD3^high^ phenotype by flow cytometry in 76 pediatric patients with pneumonia, 55 patients with severe pneumonia (SP), and 29 healthy controls (Con).

**Results:**

The numbers of circulating DNT cells were similar in all groups; however, the frequency of CD3^low^ DNT cell subsets was significantly increased in patients with pneumonia (*p* < 0.001) and SP (*p* < 0.001). The elevated CD3^low^ DNT cell frequency showed a positive correlation with the clinical severity of pneumonia. On sub-group analysis, the frequency of CD3^low^ DNT cells was only elevated in children with pneumonia aged <5 years, while no association was observed with the causative pathogen of pneumonia.

**Conclusions:**

These findings suggest that CD3 expression levels on DNT cell subsets of peripheral lymphocytes may be a valuable biomarker for evaluation of immune response in pediatric infectious disease. CD3^low^ DNT cells were elevated in children with pneumonia aged <5 years, which indicates that it may be an important research target in pediatric infectious diseases.

## Introduction

Infectious diseases are a predominant cause of death among children (Kollmann et al., 2017). T lymphocytes are shown to be associated with early life adversity wherein cell-specific adaptive immune responses are largely absent (Elwenspoek et al., 2017). Accumulating evidence suggests that CD3^+^CD4^−^CD8^−^ double-negative T (DNT) cells (i.e., CD3^+^ T lymphocytes that express neither CD4 nor CD8 surface molecules) can act as ready-made innate effector cells and may be the most important T cell subset during the first episode of infection in infancy prior to the development of adaptive immune response to pathogenic microbes (Bochennek et al., 2016; Kawano et al., 1994). DNT cells constitute approximately 1–5% of circulating T lymphocytes in healthy humans, non-human primates, and mice (Tullio et al., 2013). However, the percentage of DNT cells may rise to 10–40% of the total circulating lymphocytes in swine Influenza virus (SIV) infected natural hosts (Vinton et al., 2011; Sundaravaradan, Mir & Sodora, 2012). Tuberculosis patients with severe pathology were shown to exhibit decreased proportions of DNT cells (Pinheiro et al., 2012). Till date, no study has investigated the relationship between CD3 expression levels on DNT cells and infectious disease.

DNT cells express intermediate levels of TCR/CD3 and are mainly classified into αβ DNT and γδ DNT cells; of these, the γδ DNT cells are predominant in peripheral blood (Lai et al., 2014; Voelkl, Gary & Mackensen, 2011). Unlike αβ DNT cell, γδ DNT cells can recognize antigens directly without major histocompatibility complex (MHC) molecules and, therefore, have the ability to directly respond to specific pathogens. Therefore, γδ DNT cells readily form a bridge between the innate and adaptive immune systems (Urban, Chapoval & Pauza, 2010). Among the γδ DNT cells, the Vδ1^+^ T cell subset represents the CD3^low∕dim^ phenotype and is enriched in thymus and epithelial tissues such as gut epithelium, spleen and dermis. Vδ2^+^ T subset is characteristic with the CD3^high∕bright^ phenotype and is capable of antibody-dependent cell-mediated cytotoxicity (Bonneville, O’Brien & Born, 2010; Yi, Li & Mo, 2017; Fisher et al., 2014). Several studies have shown that Vδ2^+^ T cells expand markedly *in vivo* (up to 30% of circulating T lymphocytes) following malaria infection in previously naïve hosts (Farrington et al., 2016; Ho et al., 1990). Similarly, studies have shown that repeated malaria infection during childhood results in progressive loss and dysfunction of Vδ2^+^γδT cells (Jagannathan et al., 2014). These findings suggest that CD3^high∕bright^ DNT cells (Vδ2^+^γδT cells with CD3^bright^ phenotype) may play an important role in the control of infection and that loss or decrease of CD3 expression on DNT cells may serve as a biomarker for predicting the prognosis of pediatric patients with infectious disease.

In this study, we determined the frequency of circulating DNT cells with CD3^high^ or CD3^low^ phenotype in children with pneumonia. By analyzing the mean fluorescence intensity (MFI) of CD3 on DNT, we investigated the potential correlation between the frequency of CD3^low^ DNT cells and the clinical severity of the disease. The objective was to identify a novel biomarker for evaluation of immune response in pediatric infectious diseases by using a routine clinical test for TBNK subsets.

## Materials and Methods

### Patients and controls

Peripheral blood samples were obtained from 131 pediatric patients and 29 healthy individuals recruited at the Institute of Pediatrics, the First Hospital of Jilin University, Changchun, China, from October 2016 to April 2017. Written informed consent was obtained from all subjects prior to their enrolment. The study was approved by the Human Ethics Committee of the First Hospital of Jilin University (Ethical Approved No. 2016-405). The classification of pneumonia severity was based on the criteria defined by the World Health Organization (WHO) and established in the Brazilian Guidelines for community acquired pneumonia in Pediatrics (WHO, 2013; Sociedade Brasileira de Pneumologia e Tisiologia, 2007). The diagnosis of pneumonia was based on radiological evidence of pulmonary consolidation in combination with increased respiratory rate (respiratory rate ≥ 50 breaths per minute in children aged 2–11 months or ≥ 40 breaths per minute in children aged ≥1 year) (WHO, 2013; Sociedade Brasileira de Pneumologia e Tisiologia, 2007). Severe pneumonia was defined by severe chest indrawing in children with cough or difficult breathing or by the presence of general danger signs in patients with signs of pneumonia (inability to breastfeed or drink, lethargy or reduced level of consciousness, convulsions) (WHO, 2013; Sociedade Brasileira de Pneumologia e Tisiologia, 2007). The pediatric patient population was composed of children aged 0–15 years who were diagnosed with pneumonia (*n* = 76) or severe pneumonia (*n* = 55). Peripheral blood samples were collected from patients with pneumonia or severe pneumonia at the time of first clinical diagnosis and prior to receiving any treatment. Patients with recurrent pneumonia, those with a history of chronic pulmonary, renal, or cardiovascular disease, and those with recent surgical intervention were excluded. Data pertaining to demographic, clinical characteristics, and etiology were collected and analyzed. Subjects with immunodeficiency, autoimmune diseases, human immunodeficiency virus (HIV) infection, any infectious diseases or lung disease were excluded from the healthy control group.

### Laboratory methods

Specific etiology was determined based on the diagnostic work up at the central laboratory of the hospital using routine methods, such as quantitative-polymerase chain reaction (qPCR) and bacterial culture. Immunophenotyping of peripheral blood lymphocytes was performed using 10-colour/three laser flow cytometer (FACSCanto™; BD Bioscience, San Jose, CA, USA), BD FACSCanto™ software, and BD FACSDiva™ software (BD Bioscience, San Jose, CA, USA). Freshly collected EDTA-anticoagulated whole blood samples were collected usually on the day after the onset of fever (Table 1). Absolute counts and percentage of lymphocyte subsets were determined with a BD Multitest 6-Color TBNK reagent (CD45 PerCP-Cy5.5 clone 2D1/CD3 FITC clone SK7/ CD4 PE-Cy7 clone SK3/CD8 APC-Cy7 clone SK1/CD19 APC clone SJ25C1/CD56 PE clone NCAM 16.2+CD16 PE clone B73) using standard lysis-no-wash procedure and TRUCount tubes (BD Bioscience, San Jose, CA, USA). The lymphocyte subsets were determined by total T cells (CD3^+^), total B cells (CD3^−^CD19^+^), and total natural killer (NK) cells (CD3^−^CD56^+^/CD16^+^). The total T cells were further subdivided into subgroups by CD4 and CD8. The gating strategy for DNT cell subsets is according to the special purpose shown in different figures in the results section. The minimum cell number analyzed in DNT cells (CD3^+^CD4^−^ CD8^−^) gate was 50 cells. The CD3 expression levels on DNT, CD4^+^T, CD8^+^T, and total T cells were determined from MFI analyzed by flow cytometer.

**Table 1:** Demographic, clinical findings and aetiology were detected among participants.

	**Controls**	**Patients**		
		**Pneumonia**	**Severe Pneumonia**	*P* value
**Demographic features**				
Total number, *n*	29	76	55	
Males, *n* (%)	18 (62%)	47 (62%)	33 (60%)	0.831
**Age subgroup**				
0∼12 months, *n*(%)	10 (34.5%)	28 (36.8%)	28 (50.9%)	0.108
13 months∼5 years, *n*(%)	9 (31.0%)	28 (36.8%)	13 (23.6%)	0.108
6 ∼ 16 years, *n*(%)	10 (34.5%)	20 (26.4%)	14 (25.5%)	0.912
**Clinical findings**				
Axillary temperature in °C	36.4 ± 0.5	37.2 ± 0.9	37.4 ± 0.7	0.1718
Duration of cough in days	–	3.5 ± 1.1	3.4 ± 1.5	0.6723
Duration of difficulty breathing in days	–	1.7 ± 1.5	1.8 ± 1.3	0.6914
Duration of fever in days	–	1.8 ± 0.9	1.9 ± 0.7	0.4933
**Aetiology**				
Viral infection	–	16 (21.1%)	15 (27.3%)	0.4144
Bacterial infection	–	21 (27.6%)	13 (23.6%)	0.6901
Mycoplasma or Chlamydia infection	–	20 (26.3%)	12 (21.8%)	0.6810
Mixed infection	–	19 (25.0%)	15 (27.3%)	0.8409

**Notes.**

–, not applicable.

**Figure 1:**
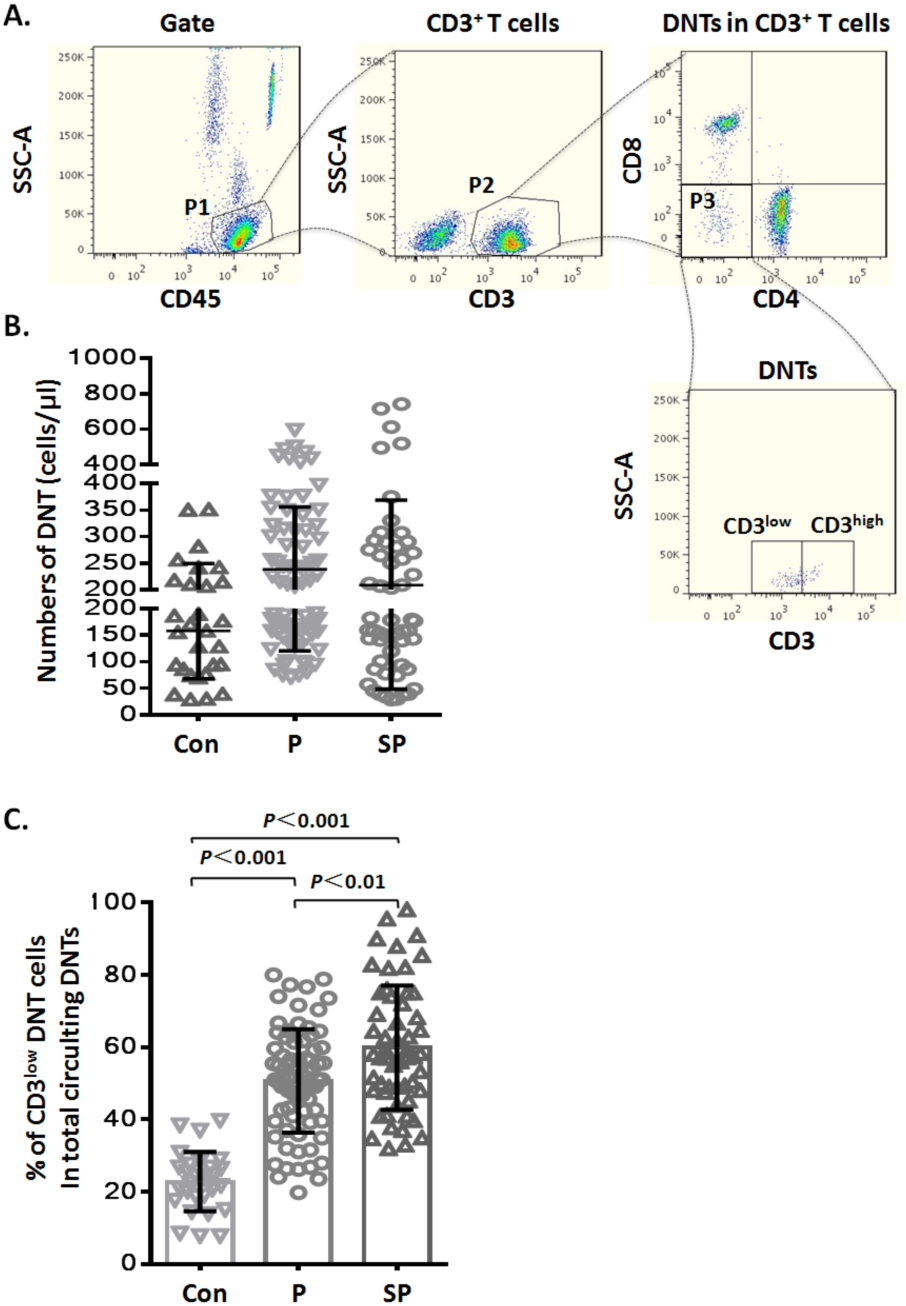
Elevated CD3^low^ DNT cells in pediatric patients with pneumonia. (A) Flow-cytometry dot plots show the strategy for gating DNT cells with CD3^low^ and CD3^high^ phenotype. (B) Numbers of DNT cells among circulating CD3^+^ T lymphocytes. (C) Percentages of CD3^low^ DNT cells among total circulating DNT cells. Con, control (*n* = 29); P, pneumonia (*n* = 76); SP, severe pneumonia (*n* = 55).

### Statistical analysis

All statistical analyses were performed using SPSS 17.0 software (SPSS Inc, Chicago, IL, USA). Clinical data are expressed as mean ± standard deviation (SD). For quantitative analysis, between-group differences with respect to normally distributed variables were assessed using Student’s *t* test while those with respect to non-normally distributed variables were assessed using non-parametric Mann–Whitney *U* test. Differences between three or more groups with respect to continuous variables were assessed using one-way analysis of variance for normally distributed variables and Kruskal–Wallis test for non-normally distributed variables. The Chi-squared test was used for qualitative analysis. A *p*-value <0.05 was considered statistically significant. Receiver operating characteristic (ROC) curve analysis was performed to assess the diagnostic performance of the biomarkers. The optimal threshold level for CD3 MFI on DNT was obtained based on the Youden index (defined as the sum of sensitivity and specificity minus one). Based on the maximal Youden index, the sensitivity and specificity were defined.

## Results

### CD3^low^ DNT cells were elevated in pediatric patients with pneumonia

During routine test of circulating TBNK subsets by flow cytometry, we noticed variable CD3 levels on DNT lymphocytes. To explore whether the CD3 expression levels on DNT cells were related to infectious disease, we analyzed the data of TBNK subsets from pediatric pneumonia or severe pneumonia patients. The general information of the patients is shown in Table 1. First, we analyzed the numbers of circulating DNT cell subsets by gating for CD45^+^CD3^+^CD4^−^CD8^−^ cells (Fig. 1A) in pneumonia patients and healthy controls; we found that the numbers of DNT cells in healthy controls were not significantly different from those in patients in the pneumonia (*p* > 0.05) and severe pneumonia (*p* > 0.05) groups (Fig. 1B). Then, we analyzed the ratios of CD3^low^ DNT cell subsets in total circulating DNT cells and found that the proportion of CD3^low^ DNT cells in peripheral blood of patients with pneumonia and severe pneumonia was significantly elevated (*p* < 0.001) and showed a positive correlation with the severity of pneumonia (*p* < 0.01) (Fig. 1C). These findings suggest that the frequency of circulating CD3^low^ DNT cell subsets may predict the severity of infectious disease, such as pneumonia.

### Age-related differences of CD3 levels on DNT cell subsets in pediatric pneumonia patients

**Figure 2:**
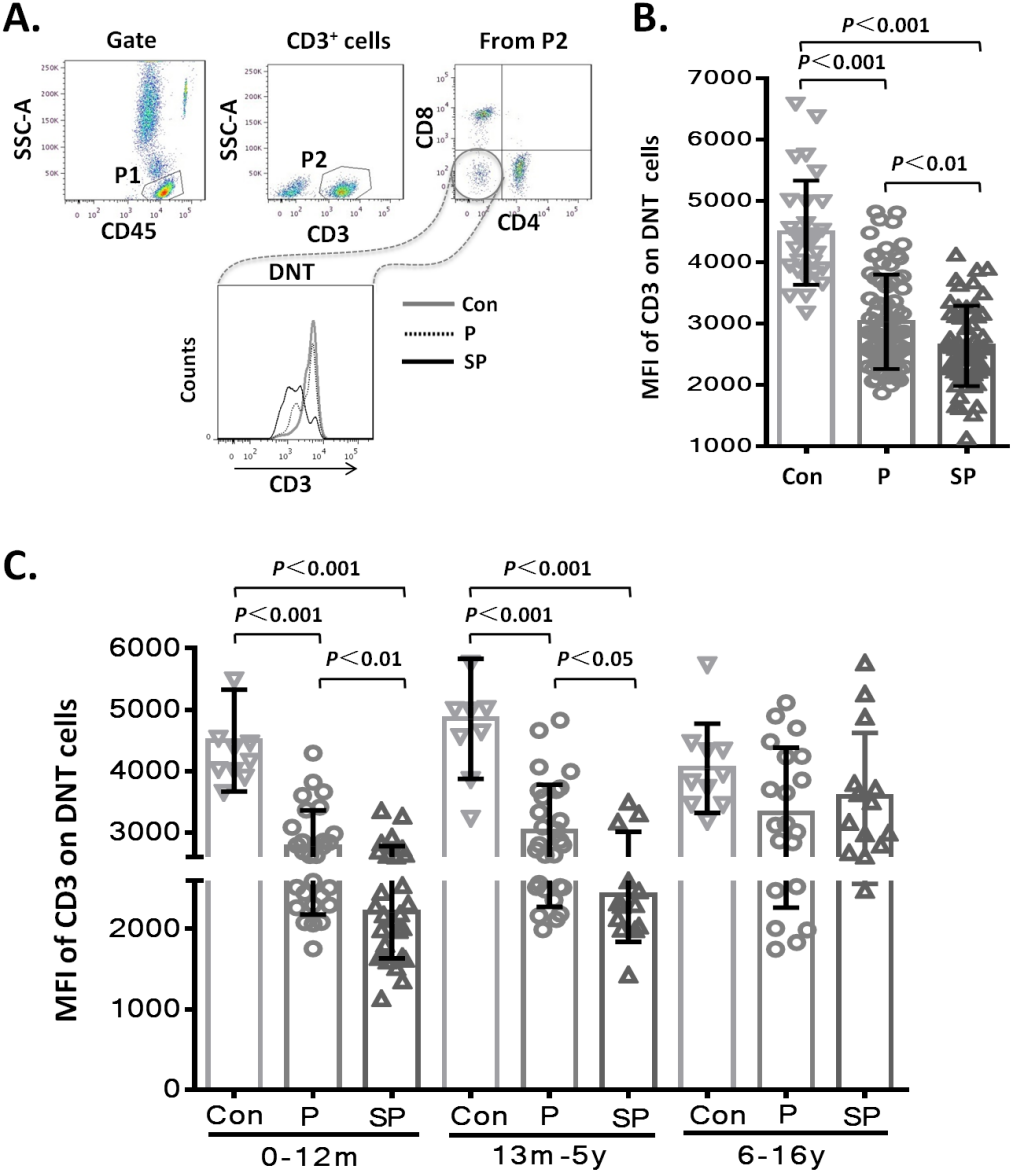
MFI of CD3 on DNT cells in pediatric pneumonia patients. (A–B) MFI of CD3 on DNT cells in different groups. (C) MFI of CD3 on DNT cells in patients in different age-groups. 0–12m: children aged 0–12 months; 13m–5y: children aged between 13 months–5 years; 6–16y: children aged between 6–16 years.

To assess whether the level of CD3 expression on DNT cell subsets showed differences with the severity of pneumonia, we first analyzed the MFI of CD3 on DNT cell subsets in the different groups. The results showed that the MFI of CD3 on DNT cell subsets in patients with pneumonia or severe pneumonia were significantly lower than that in the healthy control group (*p* < 0.001 and *p* < 0.001, respectively). Moreover, the MFI of CD3 on DNT cells from patients with severe pneumonia was significantly lower than that in patients with pneumonia (*p* < 0.01) (Fig.  2A). These findings indicate a positive correlation between the CD3 levels on DNT cell subsets and the severity of pediatric pneumonia. Then, we further investigated the effect of age on the changes in CD3 expressions levels on DNT by disaggregating the subjects into 3 age-groups: 0–12 months, 66 children (41.2%); 13 months–5 years, 50 children (31.3%); 6–15 years, 44 children (27.5%). As shown in Fig. 2B, the MFI of CD3 on DNT subsets in patients with pneumonia and severe pneumonia aged less than 5 years were significantly lower than that in control group (*p* < 0.001). Moreover, the MFI of CD3 on DNT subsets in patients with severe pneumonia was significantly lower than that in patients with pneumonia (*p* < 0.05). However, in the 6–15 year age-group, no significant differences in this respect were observed between the control, pneumonia, and the severe pneumonia groups. These results suggest that CD3 levels on DNT subsets in peripheral blood can only be used as a biomarker of clinical severity of respiratory system infection in patients aged <5 years.

In this study, none of the patients with pneumonia had concomitant diseases. In the severe pneumonia group (*n* = 55), 25 children had concomitant diseases (heart failure (*n* = 13), respiratory failure (*n* = 6), toxic encephalopathy (*n* = 3), hematuria (*n* = 2), and gastrointestinal hemorrhage (*n* = 1)). To explore the impact of concomitant disease on lymphocyte subsets, we analyzed the data of TBNK subsets from patients with severe pneumonia and found that the concomitant diseases did not affect the proportions and numbers of TBNK subsets (Figs. S1A and S1B). During analysis of the MFI of CD3 in DNT cell subsets, we further confirmed that the changes in CD3 expression levels on DNT cell subsets were not affected by concomitant diseases (Figs. S1C).

### Relationship between increased CD3^low^ DNT cell subsets and the causative pathogen

**Figure 3:**
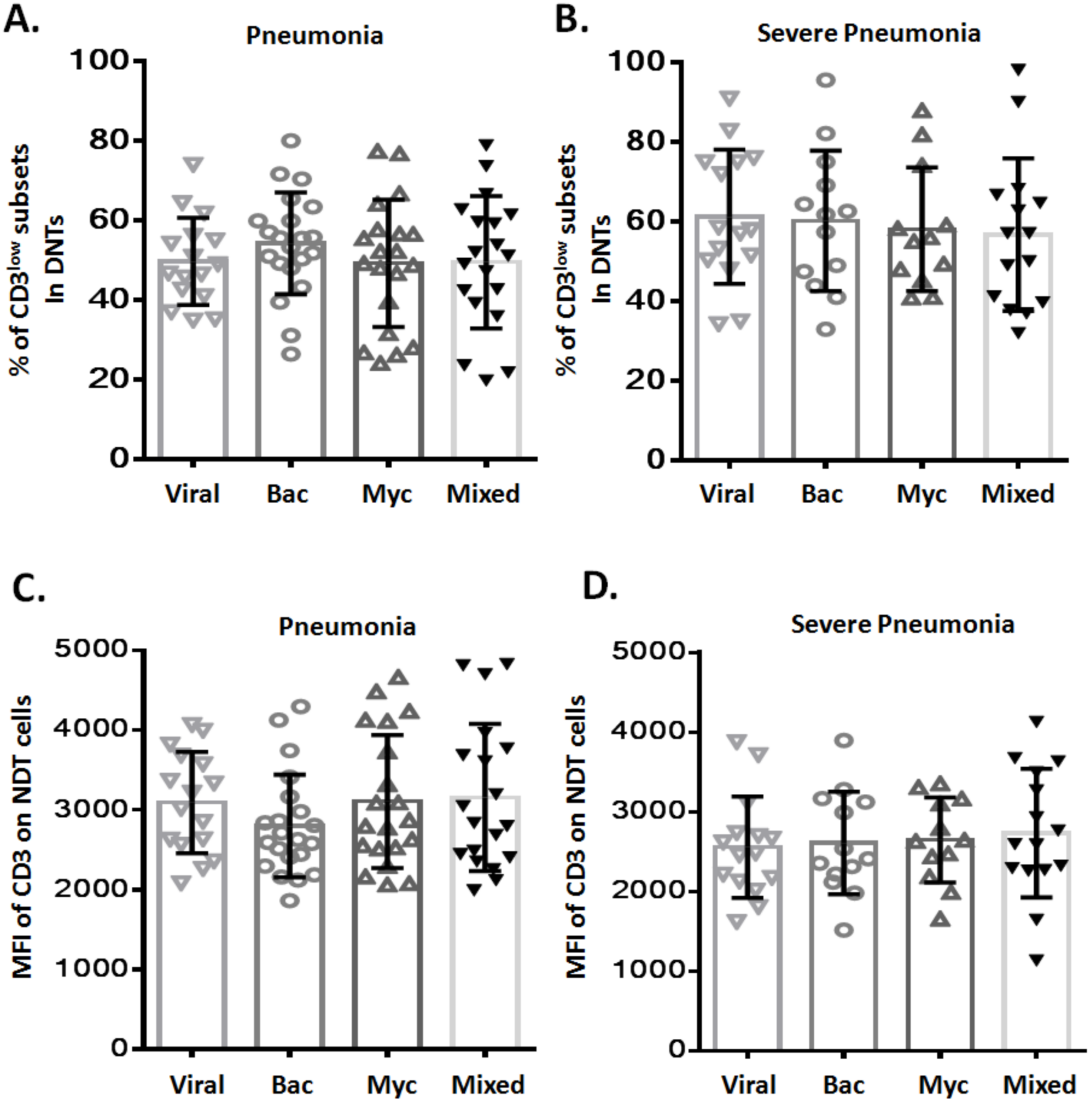
Relationship between frequency of CD3^low^ DNT cells and causative pathogen of pneumonia. The frequencies of CD3^low^ DNT cells in (A) pneumonia group and (B) severe pneumonia group. MFI of CD3 on DNT cells in (C) pneumonia group and (D) severe pneumonia group. Viral, viral infection group; Bac, bacterial infection group; Myc, mycoplasma/chlamydia pneumonia infection group; Mixed, mixed infection group.

To assess any potential relation between CD3^low^ DNT cell subsets and pathogenic profile of pneumonia, patients with pneumonia and severe pneumonia were further divided into four sub-groups based on the causative pathogen: viral infection (Viral), bacterial infection (Bac), mycoplasma/chlamydia pneumonia infection (Myc), and mixed infection (Mixed). We observed no significant difference between these four sub-groups with respect to the frequency of CD3^low^ DNT cell subsets in both pneumonia (Fig. 3A) and severe pneumonia (Fig. 3B) groups. Moreover, no significant differences were observed between the four sub-groups with respect to the MFI of CD3 in DNT subsets in both pneumonia (Fig. 3C) and severe pneumonia (Fig. 3D) groups. These findings suggest no relationship between the increased CD3^low^ DNT cell subsets in pneumonia and the causative pathogens.

**Figure 4:**
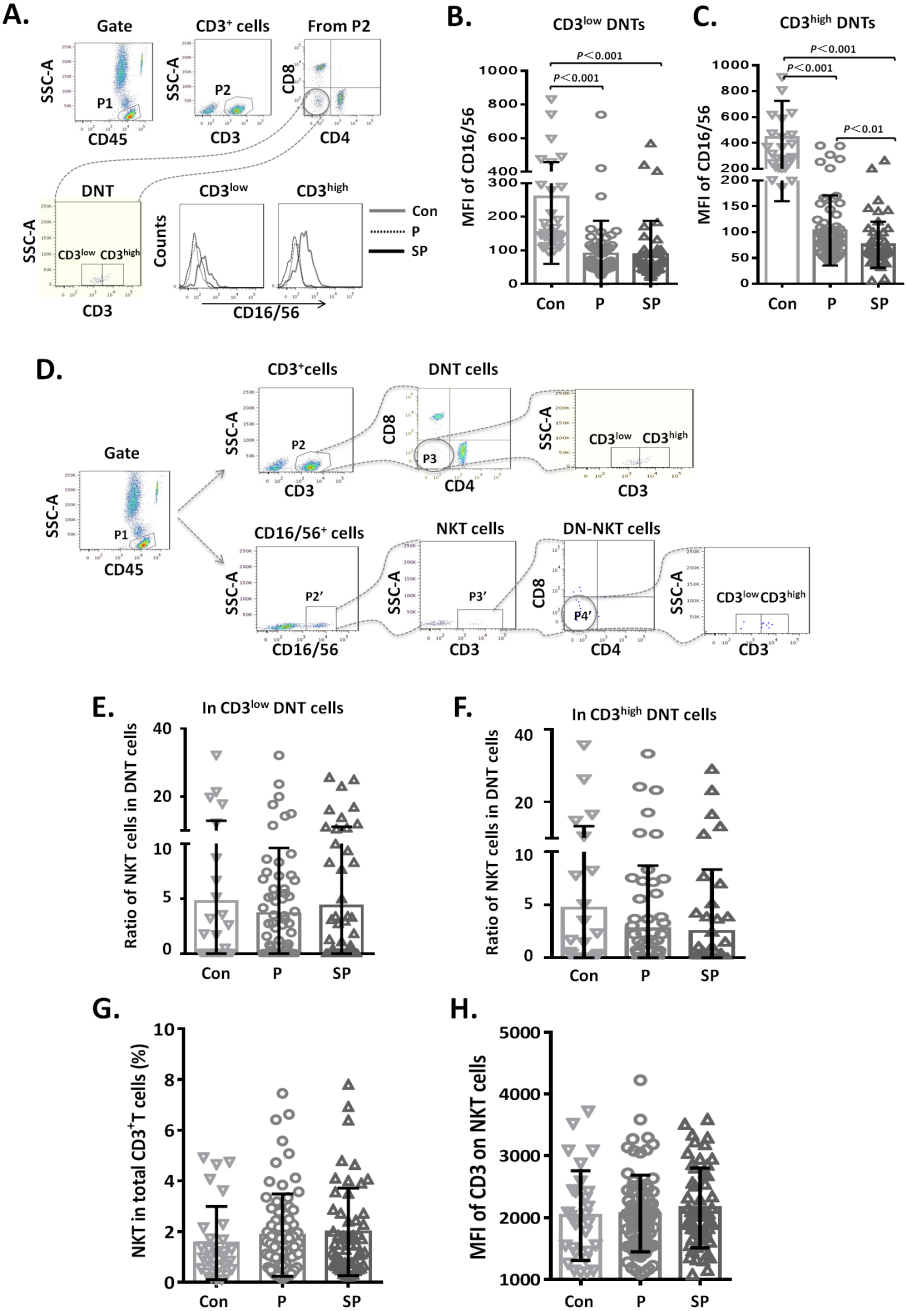
Relationship between CD3^+^CD16/56^+^ NKT cells and CD3^low^ DNT cells. (A–C) MFI of CD16 on CD3^low^ and CD3^high^ DNT cell subsets; (D–F) ratio of NKT cells in CD3^low^ and CD3^high^ DNT cell subsets. The minimum cell number analyzed in CD3^+^CD16/CD56^+^CD4^−^CD8^−^ gate was 2 cells. (G, H) Relationship between NKT and outcomes of pediatric pneumonia.

### Relationship between CD3^+^ CD16/56^+^ NKT cells and CD3^low^ DNT cells

To clarify whether the increased DNT cell subsets in pneumonia patients were NKT cells, we first analyzed the CD16/56 expression levels on DNT cells. We observed that the MFI of CD16/56 in both CD3^low^ DNT cells and CD3^high^ DNT cells in pneumonia patients was significantly lower than that in the control group (Fig. 4A). Subsequently, we determined the ratios of CD3^+^CD16/56^+^ NKT cells in CD3^low^ DNT cells and CD3^high^ DNT cells and found no significantly different between the control, pneumonia, and severe pneumonia groups in this respect (Fig. 4B). Finally, we assessed the relationship between NKT and outcomes of pediatric pneumonia. We found similar proportions of NKT cells among total T cells in the healthy control, pneumonia and severe pneumonia groups (Fig. 4C). Moreover, there were no significant differences between the three groups with respect to CD3 expression levels on NKT cells (Fig. 4C). In order to exclude the effect of CD16 expression on the results, we used CD56 monoclonal antibody to detect NKT cells in a part of patients. We found similar proportions of CD3^+^ CD56^+^ NKT cells among CD3^low^ DNT cells in the healthy control, pneumonia and severe pneumonia groups (Figs. S2A–2C). These results indicate that the increased CD3^low^ DNT cells were not NKT cells.

### Predictive ability of CD3^low^ DNT cell subsets for clinical severity of pediatric pneumonia

**Figure 5:**
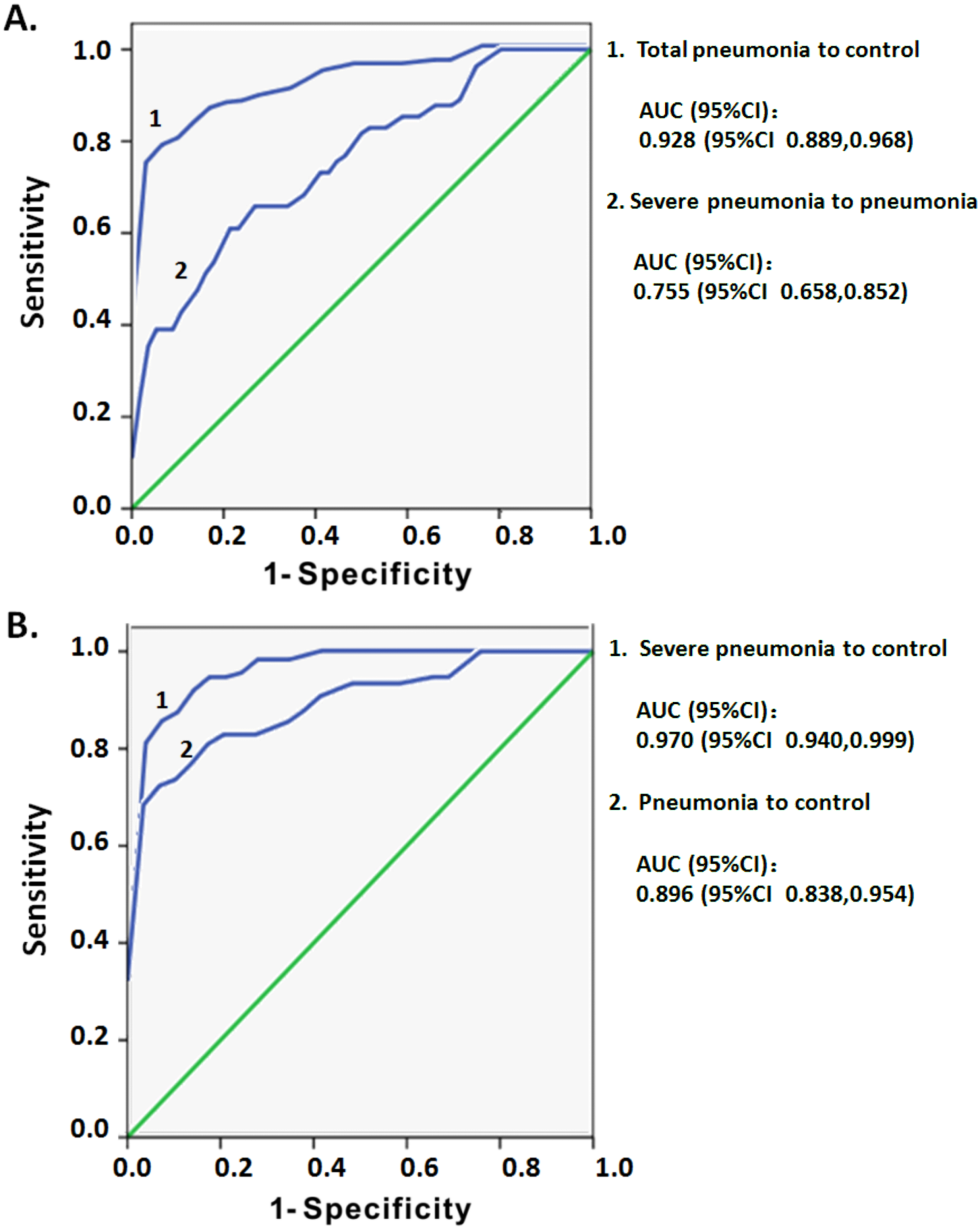
ROC curve analysis of the MFI of CD3 on DNT cells for predicting pediatric pneumonia. (A) ROC curves of the CD3 MFI of DNT cells for diagnosis of pneumonia and the severity of pneumonia. Total pneumonia: includes patients with pneumonia and severe pneumonia. (B) ROC curves of the CD3 MFI of DNT cells in severe pneumonia versus that in pneumonia.

ROC curve analysis was used to assess whether the increased CD3^low^ DNT cells in circulating lymphocytes may serve as a predictor of the severity of pediatric pneumonia. The dataset of CD3 MFI on DNT cell subsets with or without pneumonia was used to draw ROC curves. In comparing total pneumonia group (pneumonia plus severe pneumonia) and control group, the area under the curve (AUC) was 0.928 and an optimal threshold of 3,435 was associated with 78.6% sensitivity and 96.6% specificity (Fig. 5A). On subgroup analysis, the AUC for CD3 MFIs on DNT cell was 0.896 for pneumonia and 0.970 for severe pneumonia (Fig. 5B). The sensitivity and specificity to differentiate pneumonia from control were 72.4% and 96.6%, respectively, which were lower than that for differentiating severe pneumonia from control (85.5% and 96.6%, respectively). The ROC curve of CD3 MFI on DNT to differentiate severe pneumonia from pneumonia in the 0–5 year age-group had an AUC of 0.775 and the optimal threshold of 2,341.5 was associated with 61% sensitivity and 80.4% specificity (Fig. 5A). These results indicate that CD3 expression levels on DNT cell subsets in peripheral lymphocytes may be a valuable biomarker for assessment of the severity of infectious diseases, such as pneumonia.

## Discussion

Recent studies have highlighted the role of DNT cells in the systemic immune response to infection. In this study, the frequency of CD3^low^ DNT cells in pediatric patients with pneumonia was significantly higher than that in healthy controls. This finding is consistent with previous studies that demonstrated marked expansion of γδT cells (especially Vδ1^+^ γδT cells with the CD3^low∕dim^ phenotype) *in vivo* following childhood infection (Kozbor et al., 1993; Van den Heuvel et al., 2017). Previous studies have shown that there is an increased percentage of *αβ* DNT cells (another subset of DNT cells) in pediatric patients with autoimmune disease, while similar percentage of these cells were observed before and after infection (Tarbox et al., 2014; Tsutsumi et al., 2002). NKT cells with the CD3^+^CD56^+^CD16^+^ phenotype were shown to be an important subset of DNT (Khvedelidze et al., 2008). In the present study, the proportion of NKT cells among CD3^low^ DNT cell subsets did not change before and after infection. This result excludes the possibility that NKT cells affect the ratio of CD3^low^ DNT. Notably, in pediatric pneumonia, the expression level of CD16 on CD3^high^ DNT cells was significantly decreased and showed a negative correlation with disease severity. This may be attributable to the correlation between the ADCC function of CD3^high∕bright^ Vδ2^+^ γδT cells and the frequency of the CD16^+^ subset (He et al., 2012). In a previous study, decline in CD16^+^ Vδ2^+^ T cells was shown to render the Vδ2^+^ T cells incapable of ADCC response in chronic HIV-1 infection, which led to rapid disease progression (He et al., 2013). However, whether the decreased expression level of CD3 on DNT cells is related to differentiation of γδT cells or whether it is a causal event in the process of pneumonia is yet to be investigated. Nonetheless, the observed positive correlation between increased frequency of CD3^low^ DNT cells and the clinical severity of pneumonia indicates that this special T-cell subpopulation may serve as a potential marker for predicting the course of pediatric pneumonia.

Interestingly, the frequency of CD3^low^ DNT cells was only elevated in children with pneumonia aged less than 5 years. Based on the similar number of DNT, we speculate that the elevated frequency of CD3^low^ DNT cells may be closely related to the high incidence of severe pneumonia among children in the 0–5 year age-group. Several studies have shown that Vδ2^+^γδT cells with the CD3^bright^ phenotype play a role in the control of childhood malarial infection. Vδ2^+^γδT cells showed a progressive decrease with disease progression (Farrington et al., 2016; Ho et al., 1990; Jagannathan et al., 2014). These results further support our hypothesis that CD3^high∕bright^ DNT cells (Vδ2^+^ γδT cells with the CD3^bright^ phenotype) may play an important role in the control of pediatric infectious diseases and that loss or decrease in CD3 expression levels on DNT cells may serve as a biomarker for predicting the prognosis of pediatric patients with infectious diseases.

Infectious diseases are a serious threat to the health of children and recent years have witnessed a gradual increase in the associated global morbidity and mortality (Lim et al., 2017). Current strategies to control infections mainly focus on the pathogens themselves, while host factors that may regulate disease progression are largely neglected. Several studies have shown that DNT is a major responding T cell subset in human and mouse lung infection (Cowley et al., 2010; Johnson et al., 2007; Neyt, GeurtsvanKessel & Lambrecht, 2016). However, the relationship between the CD3 expression levels on DNT cells and the type of pathogen has not been investigated. In this study, we found no relation between the ratio of CD3^low^ DNT cells and the causative pathogen of pneumonia. This study suggests that CD3^low^ DNT cells may be an important research target in the field of pediatric infectious diseases.

Esposito et al. (2016) compared the sensitivity and specificity of several biomarkers to predict the severity of pneumonia. The predictive sensitivity of the assessed biomarkers ranged from 31.8–88.2%, while the predictive specificity ranged from 33.1–81.1%. In our study, we found that CD3 expression level on DNT cells may be a valuable biomarker for predicting the severity of pediatric pneumonia with a relatively good sensitivity (78.6%) and high specificity (96.6%). On ROC curve subgroup analysis, the specificity was not improved. However, the predictive sensitivity for severe pneumonia increased to 85.5%, but that for the pneumonia group decreased to 72.4%. The results further suggest that CD3 levels on DNT cells might be a more sensitive biomarker for predicting pediatric severe pneumonia. Moreover, CD3 levels on DNT cells showed a relatively good sensitivity (61%) and good specificity (80.4%) for distinguishing between severe pneumonia and pneumonia among children in the age-group of 0–5 years. Therefore, CD3 levels on DNT cells may be particular useful to predict the occurrence of severe pneumonia in children under five years of age.

## Conclusions

Taken together, our results demonstrate that CD3 expression level on DNT cell subset of peripheral lymphocytes may be a valuable biomarker for evaluation of immune responses in pediatric infectious diseases. Although the causal relationship need to be further investigated, elevated levels of CD3^low^ DNT cells were observed only in children with pneumonia aged <5 years, which indicates that it may be an important research target in pediatric infectious diseases.

##  Supplemental Information

10.7717/peerj.6114/supp-1 Supplemental Information 1 Raw data

10.7717/peerj.6114/supp-2 Figure S1 Relationship between concomitant diseases and lymphocyte subsets (A) Proportions and (B) absolute counts of total T cells (CD3^+^), B cells (CD3-CD19+) and Natural Killer (NK cells) (CD3-CD16+/CD56+). (C) MFI of CD3 on Double-negative T (DNT) cells in different groups. No: patients with severe pneumonia and no concomitant diseases (*n* = 30); Yes: patients with severe pneumonia and concomitant diseases (*n* = 25). Bars indicate the mean ±SD from independent individual subject.

10.7717/peerj.6114/supp-3 Figure S2 Relationship between CD3^+^ CD56^+^ NKT cells and CD3^low^ DNT cells (A) Flow-cytometry dot plots show the strategy for gating CD3^+^CD56^+^ NKT cells with CD3^low^ phenotype. (B) The overlapping histogram of CD56 levels were from all three subjects (Con, P and SP). (C) Ratio of CD3 ^+^ CD56^+^ NKT cells in CD3^low^ DNT cell subsets.

## Abbreviations

AUC: Area under curve
Con: Control
DNT: Double-negative T
HIV: Human immunodeficiency virus
MFI: Mean fluorescence intensity
MHC: Major histocompatibility complex
NK cell: Natural killer cell
NKT cell: Natural killer T cell
P: Pneumonia
ROC curve: Receiver operating characteristic curve
SD: Standard deviation
SIV: Swine influenza virus
SP: Severe pneumonia
WHO: World Health Organization

## Additional Information and Declarations

### Data Availability

The following information was supplied regarding data availability: The raw data are available as Supplemental Files.
