# Supplementary figures and images for: Elevated CD3low double negative T lymphocyte is associated with pneumonia and its severity in pediatric patients
Source: PeerJ. 2018 Dec 18;6:e6114. doi: 10.7717/peerj.6114 (PMC6302782; doi:10.7717/peerj.6114)

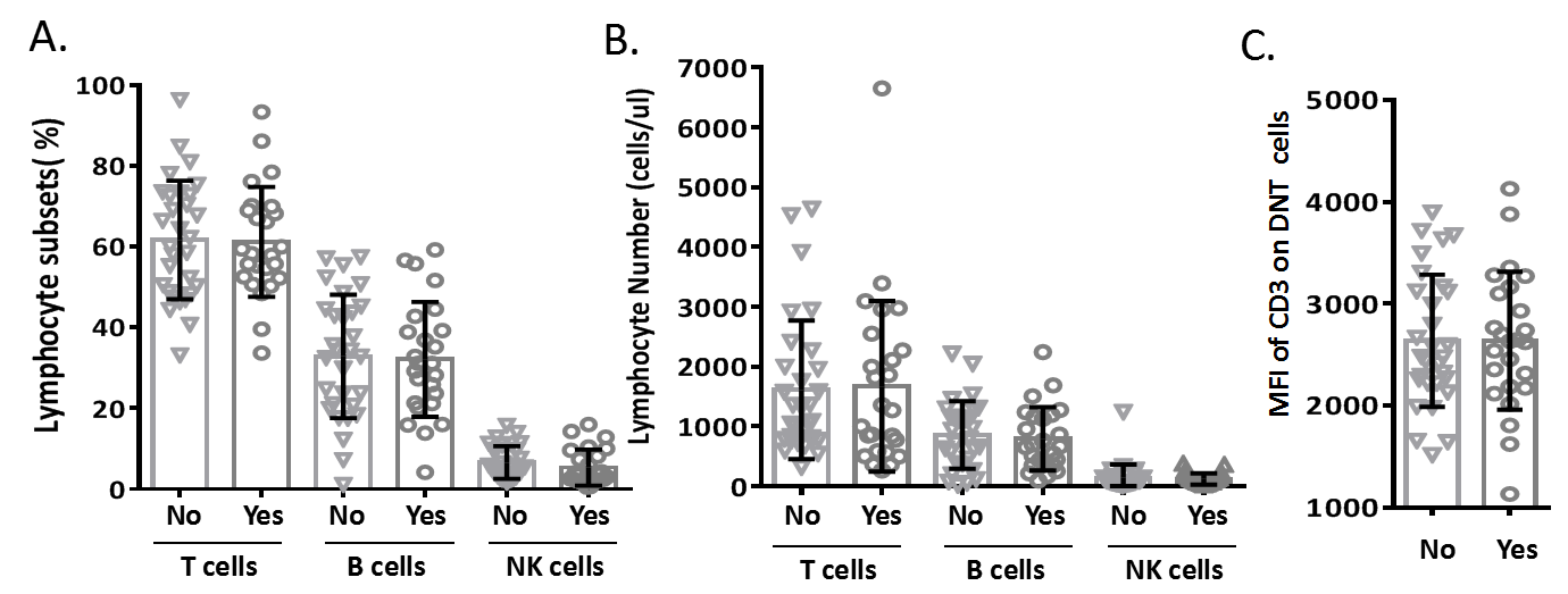

Supplement: Figure S1 — (A) Proportions and (B) absolute counts of total T cells (CD3+), B cells (CD3-CD19+) and Natural Killer (NK cells) (CD3-CD16+/CD56+). (C) MFI of CD3 on Double-negative T (DNT) cells in different groups. No: patients with severe pneumonia and no concomitant diseases (n = 30); Yes: patients with severe pneumonia and concomitant diseases (n = 25). Bars indicate the mean ±SD from independent individual subject. [file peerj-06-6114-s002.png]

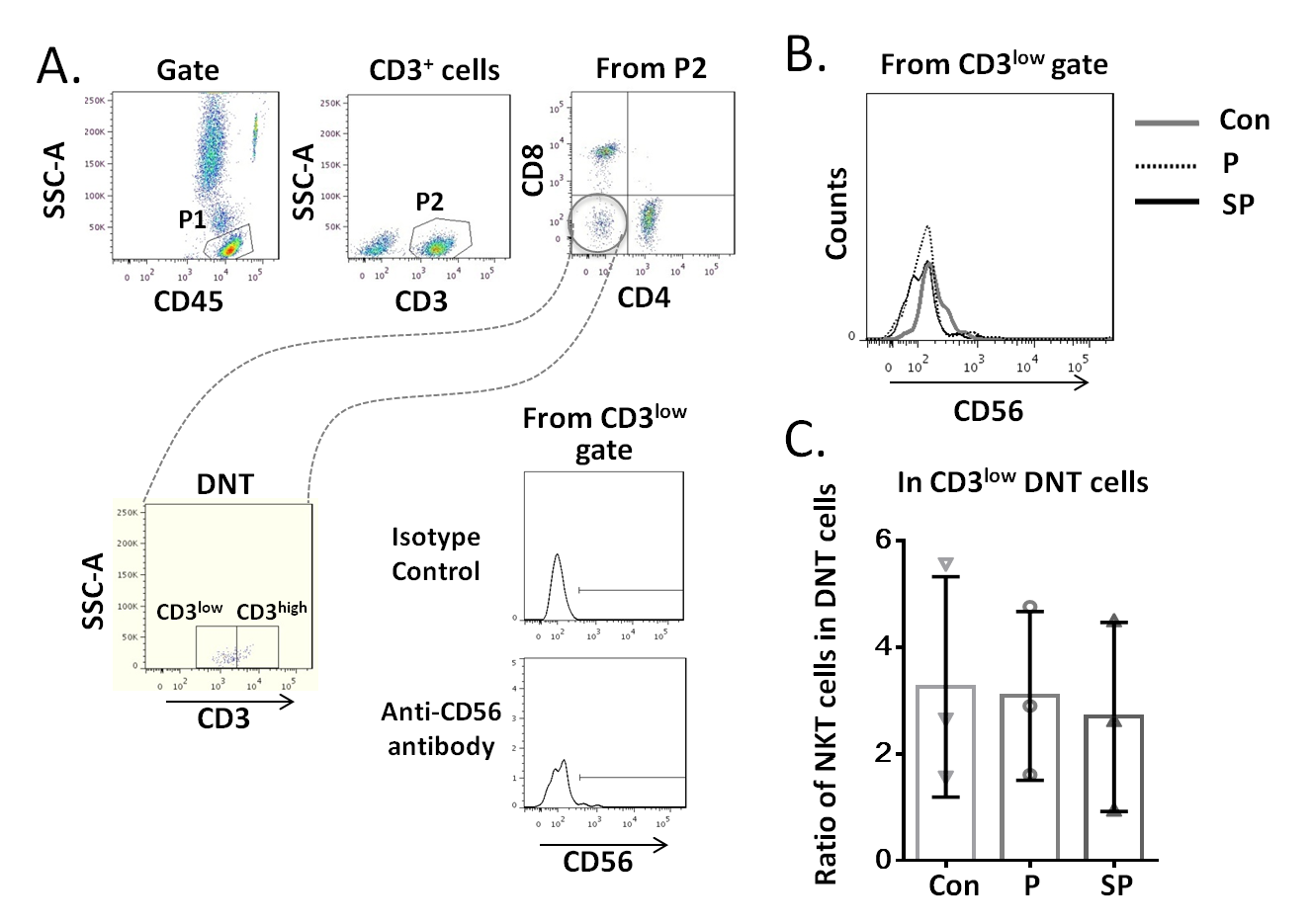

Supplement: Figure S2 — (A) Flow-cytometry dot plots show the strategy for gating CD3+CD56+ NKT cells with CD3low phenotype. (B) The overlapping histogram of CD56 levels were from all three subjects (Con, P and SP). (C) Ratio of CD3 + CD56+ NKT cells in CD3low DNT cell subsets. [file peerj-06-6114-s003.png]
